# The Numerical and Experimental Investigation of Piezoresistive Performance of Carbon Nanotube/Carbon Black/Polyvinylidene Fluoride Composite

**DOI:** 10.3390/ma16165581

**Published:** 2023-08-11

**Authors:** Kaiyan Huang, Shuying Tong, Xuewei Shi, Jie Wen, Xiaoyang Bi, Alamusi Li, Rui Zou, Wei Kong, Hui Yin, Wei Hu, Libin Zhao, Ning Hu

**Affiliations:** 1 State Key Laboratory of Reliability and Intelligence Electrical Equipment, School of Mechanical Engineering, National Engineering Research Center for Technological Innovation Method and Tool, Hebei University of Technology, Tianjin 300130, China; huangkaiyan@hebut.edu.cn (K.H.); 202111201008@stu.hebut.edu.cn (X.S.); xy_bi@hebut.edu.cn (X.B.); alamusi@hebut.edu.cn (A.L.); ruizou@cqu.edu.cn (R.Z.); lbzhao@buaa.edu.cn (L.Z.); ninghu@hebut.edu.cn (N.H.); 2 Department of Mechanical & Electronic Engineering, Sichuan Engineering Technical College, Deyang 618000, China; tongshuying@foxmail.com; 3 Xinjiang Tianfu Energy Co., Ltd., Shihezi 832000, China; shzkw@163.com (W.K.); 13677532813@163.com (H.Y.); 4 World Transmission Technology (Tianjin) Co., Ltd., Tianjin 300409, China

**Keywords:** CNT, CB, composite, piezoresistive, synergistic effect

## Abstract

The composites with multiple types of nano-carbon fillers have better electrical conductivity and piezoresistive properties as compared with composites with a single type of nano-carbon fillers. As previously reported, the nano-carbon fillers with various aspect ratios, such as carbon nanotube (CNT) and carbon black (CB), have synergistic enhanced effects on the piezoresistive performance of composite sensors. However, most of the works that have been reported are experimental investigations. The efficient and usable numerical simulation investigation needs to be further developed. In this study, based on an integrated 3D statistical resistor network model, a numerical simulation model was created to calculate the piezoresistive behavior of the CNT/CB/ Polyvinylidene Fluoride (PVDF) composite. This model also takes into account the tunneling effect between nearby nano-fillers. It is found from numerical simulation results that the piezoresistive sensitivity of composite simulation cells can be influenced by the fraction of CNT and CB. In the case that the CNT content is 0.073 wt.%, the best force-electrical piezoresistive sensitivity can be achieved when the CB loading is up to 0.2 wt.%. To verify the validity of the simulation model, the previous experimental investigation results are also compared. The experimental results confirm the validity of the model. The investigation is valuable and can be utilized to design a strain sensor for this nano-composite with increased sensitivity.

## 1. Introduction

Conductive nano-fillers/polymer composites can be applied to fabricate composite strain sensors [1,2,3,4,5,6]. Compared to the conventional metal foil strain sensor, the nano-fillers/polymer conductive composite strain sensor has the advantages of high sensitivity, high flexibility and corrosion resistance. To date, significant advances have been achieved by using CNT, CB, graphene or other nano-carbon fiber to fabricate composites strain sensors [7,8,9,10]. The physical characteristics of nano-carbon fillers have an obvious influence on the conductive performance of carbon nano-composites. Due to the variation in the microscopic conductive networks constructed by nano-carbon fillers, the resistance of the composite can change with the applied strain. For composite piezoresistive strain sensors, the methods to further improve the electrical conductivity and piezoresistive effect performance of composites are the focal point of studies. Because of the tunneling effect, carbon nano-composite strain sensors have excellent piezoresistive sensitivity. Furthermore, the influence of the fabrication process on sensitive performance is also investigated. Then, scholars try to improve the probability of the occurrence of tunneling effect to improve the piezoresistive sensitivity of carbon nano-composite strain sensors. Some novel processes that have beneficial effects on the sensing performance of composite strain sensors have been reported. Through the construction of micro-conductive networks and macro-conductive networks, sparse microscope conductive networks with more tunneling effect areas are obtained [11,12,13,14,15,16].

Recently, the synergistic effect of various types of conductive nano-fillers on the electrical properties of nano-composites has been demonstrated [17,18,19,20,21]. For instance, Ke et al. [18] and Zheng et al. [19] reported that part of CB particles is expected to bridge the gaps between CNTs when the amount of CNTs is not enough to form conductive pathways by themselves. The nano-composites with such a microscopic conductive network structure are very susceptible to strain. Lee et al. [20] reported the hybrid strain sensors featuring a piezoresistive composite made from CNT, graphene, or a combination of the two in PDMS to form the screen printable composites. The piezoresistive composites were sandwiched between PDMS layers to realize flexible strain sensors with a high gauge factor (GF) and capable of high strain level operation. Cai et al. [21] developed a kind of CB/graphene/silicon rubber composite. It is suggested that the conductivity of the composite filled with CB/graphene hybrid fillers in the mass ratio of 1:2 is much higher than that in another ratio. The synergistic effect of hybrid fillers on the electrical conductivity of composites is demonstrated. Chen et al. modified the CNT conductive network by adding SiO_2_ particles to CNT/PDMS composites [22]. Benefiting from the space occupation of SiO_2_ particles, both the electrical conductivity and piezoresistance of SiO_2_/CNT/PDMS composites were improved compared to CNT/PDMS composites.

Nevertheless, most works are based on experimental investigation. The analyses of results are not accurate enough because of the limited test samples. The numerical simulation investigation of the conductive and piezoresistive performance of carbon nano-composites is a benefit to verifying the correctness of experimental results and modified experimental schemes. In previous works, Hu et al. [23], Gong et al. [24] and Chen et al. [25] have reported some numerical studies on the electrical and piezoresistive performance of nano-carbon fillers/polymer composites. However, the numerical model for electrical characteristics of composites with hybrid nano-fillers is not complete. The numerical model still needs to be further improved in terms of accuracy, efficiency and generalizability.

In this work, a calculation scheme is proposed based on the 3D resistance networks model to simulate the conductivity and piezoresistivity of CNT/CB/polymer composites. The appropriate nano-carbon filler content and proportion can be forecasted relatively accurately by the numerical simulation model. Furthermore, the CNT/CB/PVDF composite samples are prepared, and their conductive performance and piezoresistive performance are tested. The experimental results prove the availability and accuracy of the numerical simulation model.

## 2. The Numerical Simulation Scheme

### 2.1. Construction of Resistor Network Model

It can be confirmed from previous work that nano-carbon fillers are randomly distributed in the polymer matrix. CNTs are treated as cylindrical conductors, and CBs are treated as spheriform conductors. For improving the efficiency of calculation, the aggregation and deformation of nano-carbon fillers are ignored. The detailed process of numerical simulation is shown as follows.

**Figure 1:**
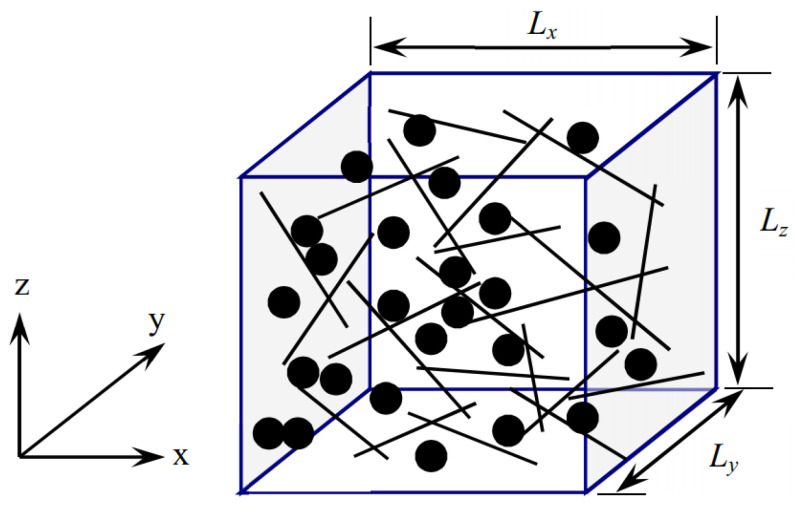
3D numerical calculation cell with CNTs and CBs.

① The simulation area is set as a cubic cell, as shown in Figure 1. The size of the cubic cell is varied for achieving a converged and stable conductive and piezoresistive performance of the calculation cell.

② CBs and CNTs are randomly put (one by one) into the 3D cube, and their orientations in the calculation cell are determined randomly. The coordinates of two ends of a randomly dispersed CNT, i.e., (*x*_1_; *y*_1_; *z*_1_) and (*x*_2_; *y*_2_; *z*_2_), can be set as Equation (1). The coordinates of the center of dispersed CB, i.e., (*x*_3_; *y*_3_; *z*_3_), can be set as Equation (2).

(1) $$\begin{array}{c} x_{1}=rand\times L_{x},\;y_{1}=rand\times L_{y},\;z_{1}=rand\times L_{z},\\ x_{2}=x_{1}+L\cdot v_{1}\cdot cos\left(w_{1}\right),\;y_{2}=y_{1}+L\cdot v_{1}\cdot sin\left(w_{1}\right),\;z_{2}=z_{1}+L\cdot u_{1},\\ u_{1}=1.0\text{–}2.0\times \mathrm{rand},\;v_{1}=\sqrt{1.0-u_{1}^{2}},\;w_{1}=2\pi \times \mathrm{rand} \end{array}$$

*x*_3_ = rand × *L_x_*, *y*_3_ = rand × *L_y_*, *z*_3_ = rand × *L_z_*. (2)

where *L_x_*, *L_y_* and *L_z_* are the lengths of the 3D element along *x*, *y* and *z* axes, respectively, as shown in Figure 1, rand is a random number located in [0, 1], which is uniformly generated. Furthermore, the parameters representing alignment directions of CNTs are expressed as *u*_1_, *v*_1_ and *w*_1_.

Some CNTs may be partially located outside of the 3D element. In this case, by looking for the intersection of these CNTs with the boundary planes of the 3D element, the part of these CNTs that are located outside of the 3D element will be automatically removed. The intersections on the boundary planes are numbered as the endpoints of these CNTs.

③ The relative location of CNTs and CBs needs to be confirmed. When a CNT or a CB particle is added to the unit cell, it is checked if it is in contact with another CNT or CB particle already present in the unit cell. It is accomplished by determining the minimum distance between the nano-carbon fillers in question and the other remaining nano-carbon fillers. When two carbon particles are found to be in contact, the intersection is numbered. Until the amount of added nano-carbon fillers reach the required percolation threshold and all intersections among CNTs and CBs are numbered sequentially to form a global conductive network.

④ Based on the well-known Kirchhoff s current law, the total current I under a certain applied voltage can be calculated. Then, the macroscopic electrical conductivity of the 3D numerical calculation cell can be evaluated using Ohm’s law. The conductivity change in CNTs and CBs under elastic strain is ignored since its contribution can be considered to be insignificant under a small strain. Furthermore, very limited deformation is expected in the CNTs and CBs due to the poor stress transfer from the matrix to the CNTs and CBs, caused by the large elastic mismatch between the nano-carbon fillers and the polymer and the weak interface strength.

In the 3D element, it is the key word to know if any dispersed CNTs and CBs are in contact to form possible conductive networks. Three situations are considered, including CNT vs. CNT, CNT vs. CB and CB vs. CB.

In this study, CNTs are regarded as cylinders of length = *L*_CNT_ and diameter = *D*_CNT_, and CBs are regarded as spheres of diameter = *D*_CB_. As shown in Figure 1, considering CNT_1_ and CNT_2_, the two ends of CNTs are marked as 1#, 2#, 3# and 4#. The coordinates of them are represented by (*x*_1_, *y*_1_, *z*_1_), (*x*_2_, *y*_2_, *z*_2_), and (*x*_3_, *y*_3_, *z*_3_), (*x*_4_, *y*_4_, *z*_4_), respectively. The direction vectors of CNT_1_ and CNT_2_, i.e., *d*_1_ and *d*_2_, are determined by Equation (3).

***d*_1_** = (*x*_2_ − *x*_1_, *y*_2_ − *y*_1_, *z*_2_ − *z*_1_), ***d*_2_** = (*x*_4_ − *x*_3_, *y*_4_ − *y*_3_, *z*_4_ − *z*_3_). (3)

In addition, the difference in coordinates of the starting points of the CNT_1_ and CNT_2_ is expressed as ***P*_13_**, shown in Equation (4).

***P*_13_** = (*x*_1_ − *x*_3_, *y*_1_ − *y*_3_, *z*_1_ − *z*_3_) (4)

**Figure 2:**
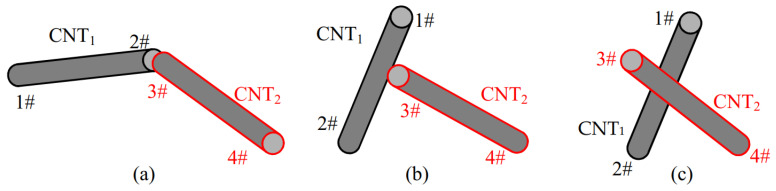
The 3 contact cases between two CNTs in 3D numerical calculation cell.

Then, the contact condition between two CNTs can be concluded as three possible situations, as shown in Figure 2. Firstly, the distances between the endpoints of CNTs, i.e., (1#, 3#), (2#, 4#), (1#, 4#) and (2#, 3#), are calculated. If the distance is smaller than *D*_CNT_, the corresponding two CNTs are considered as in contact, as shown in Figure 2a. Secondly, if none of the CNTs met the above requirement and are not parallel to each other, the four shortest distances from points 1# and 2# to CNT_2_, and from points 3# and 4# to CNT_1_, are calculated, as shown in Figure 2b. For instance, the shortest distance $d_{3\#CNT1}$ between CNT_1_ and point 3# can be simply calculated using Equation (5).

(5) $$d_{3\#CNT1}=\frac{\left\|a\times d_{1}\right\|}{\left\|d_{1}\right\|}$$

where $a=(x_{3}-x_{2},y_{3}-y_{2},z_{3}-z_{2})$, and $d_{1}$ is evaluated by Equation (3). If one of the four distances was smaller than *D*_CNT_, the two CNTs were in contact, and the corresponding pattern is illustrated in Figure 2b. Finally, we considered the situation where the two CNTs skewed off each other, as shown in Figure 2c. Again, using the coordinates of the two ends of the CNTs, the possibility of an intersection between the two CNTs can be examined. The shortest distance $d_{CNT12}$ (the length of the common perpendicular to two lines) between CNT_1_ and CNT_2_ was estimated as shown in Equation (6).

(6) $$d_{CNT12}=\frac{\left\|P_{13}\cdot \left(d_{1}\times d_{2}\right)\right\|}{\left\|d_{1}\times d_{2}\right\|}$$

If $d_{CNT12}$ is less than *D*_CNT_, then CNT_1_ and CNT_2_ are considered to be in contact, and the intersection is marked as a node.

**Figure 3:**
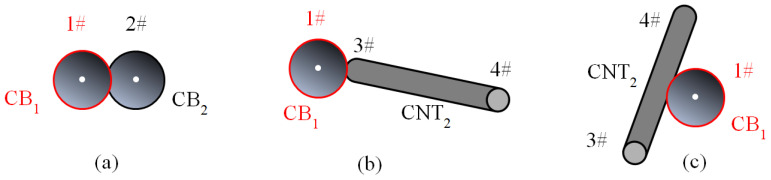
The 3 contact cases between CB and another nano-filler in the 3D numerical calculation cell.

Similarly, the contact cases between two CBs and between CB and CNT can be summarized into 3 cases, as shown in Figure 3. The two CB particles in the space are marked as CB_1_ and CB_2_. Their spherical center point coordinates are set to 1#(*x*_1_, *y*_1_, *z*_1_) and 2#(*x*_2_, *y*_2_, *z*_2_). A referenced CNT is marked as CNT_2_. The two ends of CNT_2_ are marked as 3# and 4#. The coordinates of them are represented by (*x*_3_, *y*_3_, *z*_3_) and (*x*_4_, *y*_4_, *z*_4_). The distance between CB_1_ and CB_2_ (*d*_CB12_) can be expressed by the distance between the two spherical centers 1#(*x*_1_, *y*_1_, *z*_1_) and 2#(*x*_2_, *y*_2_, *z*_2_). If *d*_CB12_ is smaller than *D*_CB_, the two corresponding CBs are considered in contact, as shown in Figure 3a. The center points of CB_1_ or CB_2_ is marked as a node. The contact situation between CB_1_ and CNT_2_ can be divided into two cases. Firstly, we calculate the distance between the center point of CB1 and the endpoints of CNT_2_ (*d*_CB1CNT2_), i.e., (1#, 3#) and (1#, 4#). If *d*_CB1CNT2_ is smaller than 0.5*D*_CB_ + 0.5*D_CNT_*, the CB_1_ and CNT_2_ are considered in contact, as shown in Figure 3b. The center point of CB1 or the contact end point of CNT_2_ is marked as a node. If the above case is not satisfied, the distance between CB_1_ and CNT_2_ is considered for the case of intermediate contact between 1# and CNT_2_. Similarly to *d*_2_ of CNT_2_ in the previous section, *d*_1#CNT_ is calculated by Equation (5). If *d*_CB1CNT2_ is smaller than 0.5*D*_CB_ + 0.5*D*_CNT_, the CB_1_ and CNT_2_ are considered as in contact, as shown in Figure 3c. The center point of CB_1_ is marked as a node.

**Figure 4:**
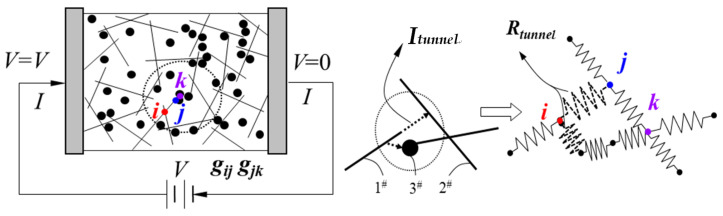
The schematic diagram of the 2D resistor network model used to calculate the conductivity of the CNT/CB/polymer composite.

CNTs and CBs are gradually generated in the calculation cell until the content of added nano-fillers reaches the percolation threshold. All the marked nodes are sequentially numbered to form a global conducting network. It is noteworthy that with the loading of CNT and CB, there is always a quasi-contact situation between nano-carbon fillers. According to Lennard-Jones potential and Van der Waals force theory, in this study, we define the occurrence distance of the quasi-contact state as 0.5 nm. In the quasi-contact state, a virtual resistance is generated between the two nodes, i.e., tunneling resistance (*R*_tunnel_). To facilitate the explanation, the 3D numerical calculation cell shown in Figure 1 is simplified to the 2D model, as shown in Figure 4.

The two electrodes of the model are connected to the power source. The total current (*I*) is calculated after the loading voltage (*V*), according to Ohm’s law. Then, the macroscopic conductivity of the 2D model can be defined as Equation (7):

(7) $$\sigma_{\mathrm{com}}=\frac{I}{V}\frac{L_{\mathrm{com}}}{S}$$

where *L*_com_ is the distance between the two electrode plates. *S* is the cross-sectional area of the calculated cell of the composite. For the conductance between nodes *i* and *j* can be defined as Equation (8):

(8) $$g_{ij}=\frac{1}{R_{ij}}=\sigma_{C}\frac{S_{C}}{l_{ij}}$$

where *l_ij_* is the length between nodes *i* and *j*, and equally so for node *k*. $\sigma_{C}$ is the electrical conductivity of nano-carbon fillers. $S_{C}$ is the cross-sectional area of the nano-carbon fillers. In this study, for CNT, $S_{C}$ is the area of the cylinder base. For CB, $S_{C}$ is the area of the great circle of the sphere. At this point, the current at *i* can be expressed as Equation (9), based on Kirchhoff’s current law.

(9) $$I_{i}=\sum_{j=1}^{N_{i}}g_{ij}\left(V_{i}-V_{j}\right)=0$$

where *V_i_* is the potential of *i*. *V_j_* is the potential of *j*. Traversing all calculation nodes and summing up the node currents connected to the pole plate, the total current *I* can be obtained. Then, the conductivity and resistance of the composite calculation cell can be obtained according to Equation (7).

**Figure 5:**
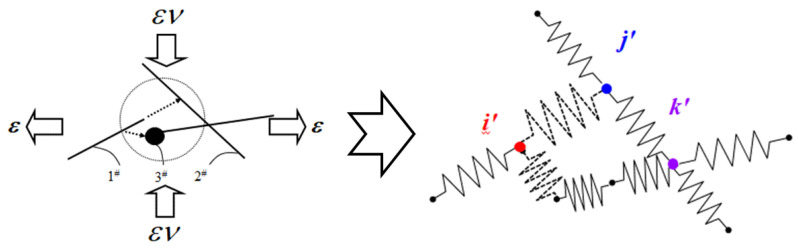
The schematic diagram of the 2D resistor network model with strain loading.

Because the elastic modulus of nano-carbon fillers is much higher than the polymer matrix, we treat CNTs and CBs as rigid bodies. The resistance change in CNTs and CBs under elastic strain is ignored since its contribution can be considered to be insignificant under a small strain. The internal resistance networks are changed, and the new position coordinates of CNTs and CBs are recalculated when the numerically calculated cell is deformed by a load, as shown in Figure 5. In the new resistance networks, the nodes information and tunneling resistance are updated. Then, the resistance of the numerically calculated cell is iteratively calculated again based on Kirchhoff’s current law.

### 2.2. The Results of Numerical Simulation Tests

**Figure 6:**
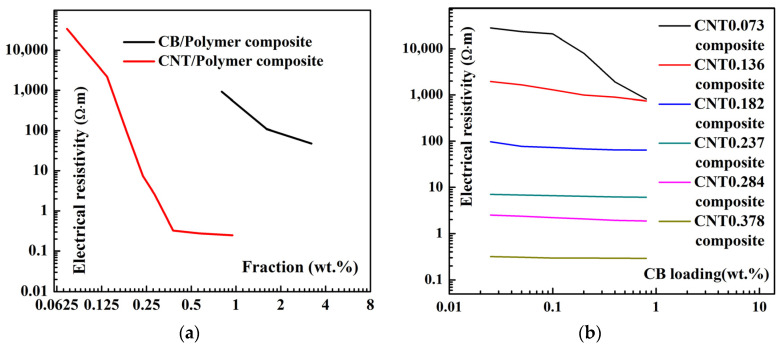
Electrical resistivity of the numerically calculated cells: (**a**) with one type of nano-carbon fillers; (**b**) with two types of nano-carbon fillers.

To verify the availability of the numerical calculation method, we first estimate the electrical conductivity of composites numerically calculated cells without applied strains. Theoretically, a stable conductive network can be formed when sufficient nano-carbon fillers are randomly generated in the polymer matrix. Reducing the size of the cell elements as much as possible can improve the efficiency of the computation while ensuring convergence of the results. Based on previous works, the dimensions of the calculation area for this example are set as *L_x_* = 5*L_CNT_*, *L_y_* = 3*L_CNT_*, and *L_z_* = 3*L_CNT_* [26]. The electrical resistivity of the numerically calculated cell with various carbon nano-filler content by the model (average of 5 tests) is shown in Figure 6.

**Figure 7:**
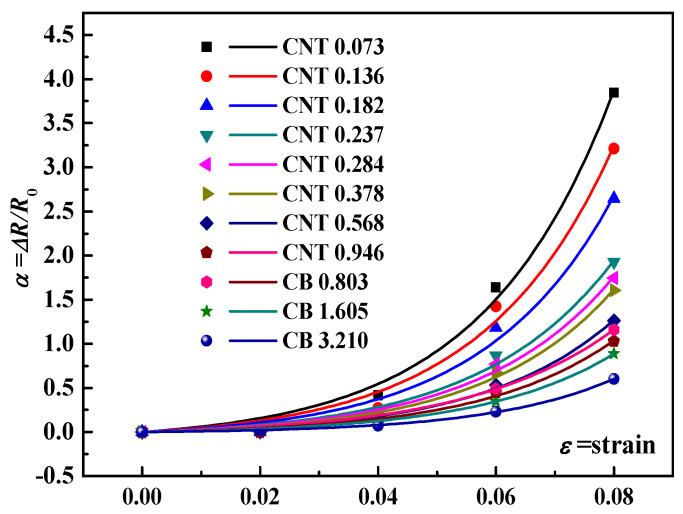
Resistance change rate (Δ*R*/*R*_0_)–strain curves for various nano-composites samples with one type of nano-carbon fillers loading.

For convenience, we have indicated the various samples in the form of nano-fillers numbers; i.e., CNT0.073 indicates the sample with 0.073 wt.% CNT. As shown in Figure 6a, the electrical resistivity decreases as the mass fraction of nano-carbon fillers in the numerically calculated cell increases. As shown in Figure 6b, the resistivity of the numerically calculated cell decreases significantly with continued loading of the CB when the CNT content is 0.073 wt.%. However, the reduction in electrical resistivity of the numerically calculated cell is no longer significant as the CB continues to be loaded when the CNT content increase. That is, at a low CNT content, CB can provide a significant synergistic effect on the conductivity enhancement of the arithmetic cytosol. Because of the sparse conductive network, a small amount of conductive fillers can significantly increase the density of the conductive network and reduce the overall resistivity of the numerically calculated cells. Then, the piezoresistive properties of the numerically calculated cells are tested by the model. Apply tensile strain *ε* to the x-axis direction of the numerically calculated cell to obtain the resistance *R_x_* after loading, and calculate the resistance change rate Δ*R*/*R*_0_, i.e., (*R_x_* − *R*_0_)/*R*_0_, where *R*_0_ is the original resistance of the numerically calculated cell. The Δ*R*/*R*_0_~*ε* relationship for single-type nano-carbon fillers loaded with the numerically calculated cell is shown in Figure 7. The simulation results show that the slope of the Δ*R*/*R*_0_~*ε* relationship curve is lower as the CNT or CB content increases; i.e., the piezoresistive sensitivity of the composite is decreased.

When the conductive network is sparse, more tunneling resistance (*R_tunnel_*) is present in the conductive network, and the presence of Rtunnel is one of the reasons for the high sensitivity of carbon nano-composites. As the nano-carbon filler content increases, the conductive network becomes increasingly dense, and the Rtunnel is shielded by the formed conductive pathway. Therefore, to obtain high sensitivity, the content of nano-carbon fillers is generally chosen around the percolation threshold. Furthermore, the number of broken conductive pathways is also an important factor for the sensitivity of carbon nano-composites. The sparse conductive network has a low number of conductive paths and a high ratio of broken conductive paths with strain loading.

**Figure 8:**
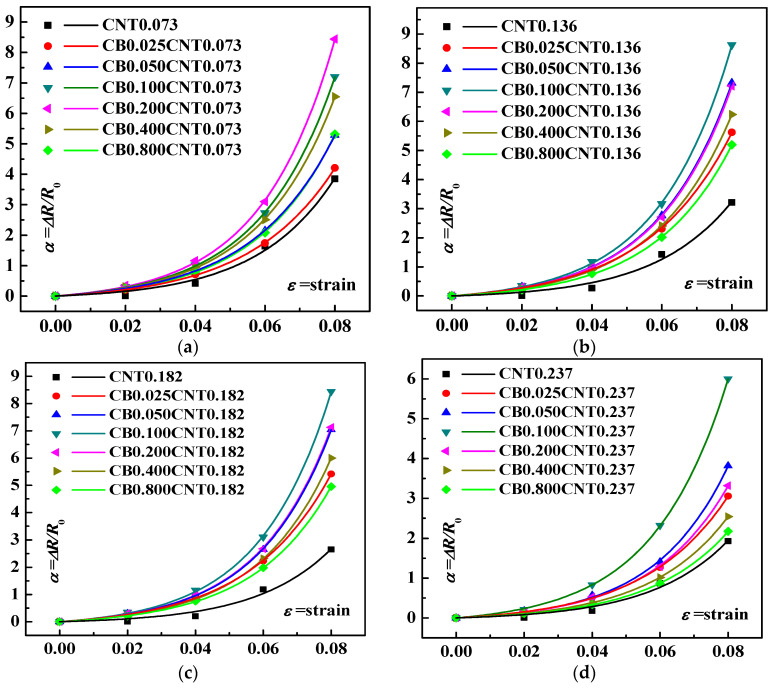

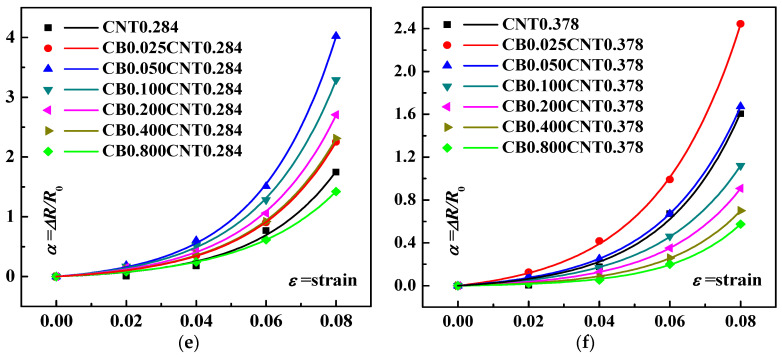
Resistance change rate (Δ*R*/*R*_0_)–strain curves for various nano-composite simulation cells: (**a**) 0.073 wt.% of CNT and varying CB loading; (**b**) 0.136 wt.% of CNT and varying CB loading; (**c**) 0.182 wt.% of CNT and varying CB loading; (**d**) 0.237 wt.% of CNT and varying CB loading; (**e**) 0.284 wt.% of CNT and varying CB loading; (**f**) 0.378 wt.% of CNT and varying CB loading.

To investigate the piezoresistive synergistic effect of CNT and CB, the Δ*R*/*R*_0_*~ε* curves are illustrated in Figure 8. Tensile strain *ε* is applied in the x-axis direction to the example cell with CNT and CB. The resistance *R_x_* of the loaded example cell can be calculated, and the resistance change rate Δ*R*/*R*_0_ can also be calculated, i.e., (*R_x_* − *R*_0_)/*R*_0_, where *R*_0_ is the original resistance of the numerically calculated cell.

Figure 8a–f show the effect of changing the CB content on the piezoresistive response of the operator cell for CNT contents of 0.073 wt.%, 0.136 wt.%, 0.182 wt.%, 0.237 wt.%, 0.284 wt.% and 0.378 wt.%, respectively. It can be seen that the sensitivity of the piezoresistive response exhibits a rising and then decreasing process as the CB content increases when the CNT content is fixed. The sensitivity of the piezoresistive response increases slowly with CB loading and reaches the best sensitivity at the point with suitable CB content when CNT content is low. For example, the best piezoresistive sensitivity is achieved when the CNT content is 0.073 wt.%, and the CB content rises to 0.2 wt.%.

**Figure 9:**
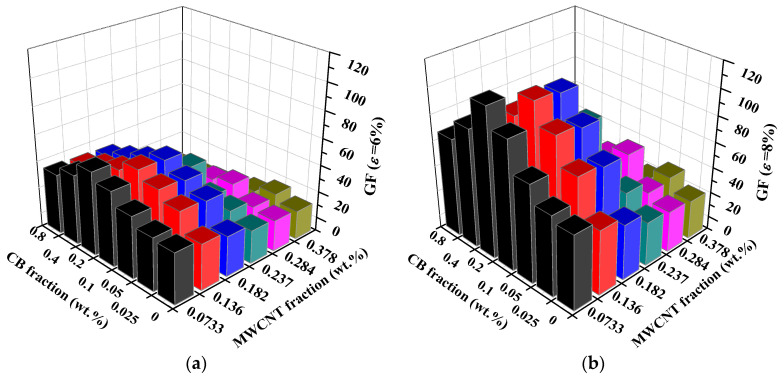
The GF value of CNT/CB calculating example cell at 6% (**a**) and 8% (**b**) strain level.

However, as the CNT content increases, the best piezoresistive response sensitivity of the composite simulation cell is achieved at a low CB content. The best piezoresistive response sensitivity for this group of tests is achieved at a CNT content of 0.378 wt.% and CB content of only 0.025 wt.%. To provide a more intuitive view of the effect of the CNT and CB content on the piezoresistive response sensitivity of the simulation cell, the GF results at 6% and 8% strain are illustrated in Figure 9.

## 3. The Results of Experimental Tests

**Figure 10:**
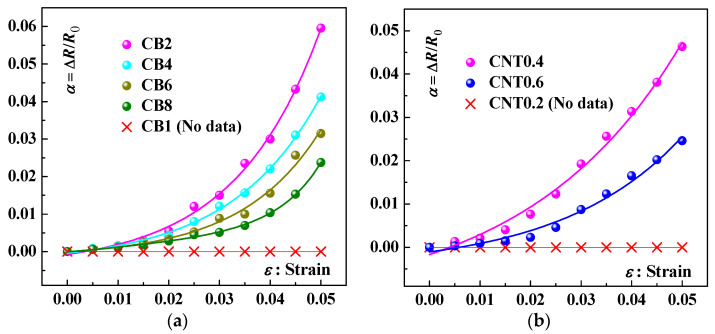
Resistance change rate (Δ*R*/*R*_0_)–strain curves for various nano-composites samples: (**a**) with varying CB loading; (**b**) with varying CNT loading.

**Figure 11:**
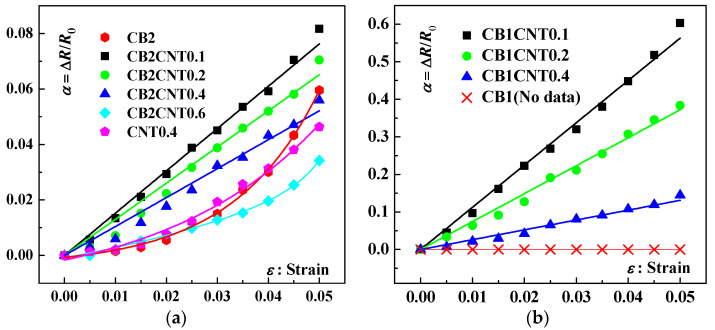
Resistance change rate (Δ*R*/*R*_0_)–strain curves for various nano-composite samples: (**a**) 2.0 wt.% of CB and varying CNT loading; (**b**) 1.0 wt.% of CB and varying CNT loading.

To verify the rationality of the numerical simulation model, the CNT/CB/PVDF composite samples were prepared via the evaporative solvent molding process based on our previous research [27]. The piezoresistive performance of composite sensors was tested and shown in Figure 10 and Figure 11. Figure 10 shows the piezoresistive response curves of tested samples with single nano-carbon fillers addition in the strain range of 0 to 0.05. The piezoresistive response curves of tested samples with various masses are shown in Figure 10a. It can be seen that the sensing signal can be measured when the CB content reaches 2 wt.%. The piezoresistive sensitivity of the test sample decreases as the fractions of CB continue to increase. Similarly, Figure 10b shows the piezoresistive response curves of tested samples with various CNT fractions. It can be seen that the sensing signal can be detected when the CNT content reaches 0.4 wt.%. The piezoresistive sensitivity of the test samples decreases as the CNT content increase.

Figure 11 illustrates the piezoresistive response curves of tested samples with dual-component nano-carbon fillers addition in the strain range of 0 to 0.05. Figure 11a shows the results of the piezoresistive response of the composite samples with a CB content of 2 wt.% and CNT content of 0.1 wt.%, 0.2 wt.%, 0.4 wt.% and 6 wt.%, respectively. It can be seen that when the CB content is 2 wt.%, only 0.1 wt.% CNT addition can significantly increase the sensitivity of the piezoresistive response of the tested samples. When the CNT content continued to increase, the piezoresistive sensitivity of the test samples then decreased. The CB content of the test sample was reduced to 1 wt.%, and the piezoresistive performance continued to be tested, as shown in Figure 11b. Similarly to the results demonstrated in Figure 11a, the highest piezoresistive sensitivity response of the tested samples was observed at 0.1 wt.% CNT content. As the CNT continues to be added, the sensitivity of the force-voltage resistance response of the tested samples gradually decreases. Notably, comparing the piezoresistive response between the sample CB2/CNT0.1 and sample CB1CNT0.1, sample CB1CNT0.1 has much more sensitive piezoresistive response sensitivity than sample CB2CNT0.1.

**Figure 12:**
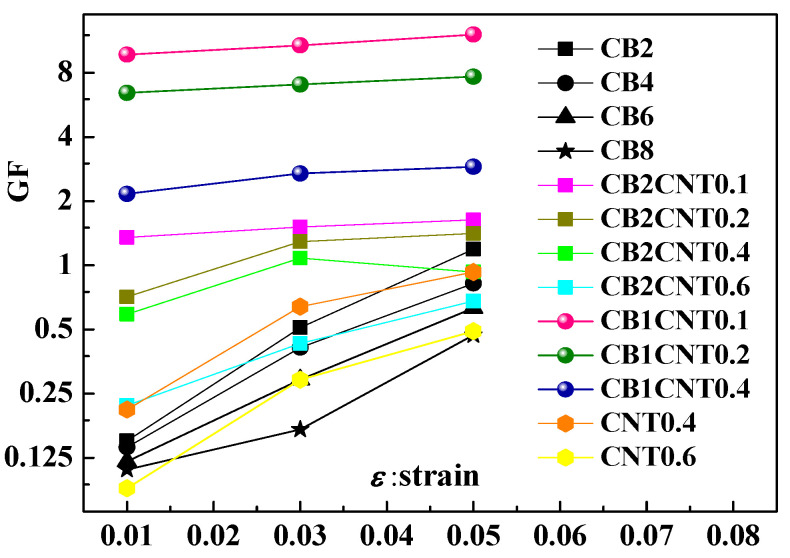
The comparison of piezoresistive effect GF of test samples.

Similarly to the numerical simulation investigation, the GF results at 1%, 3% and 5% strain are illustrated in Figure 12. It can be visualized that the test samples with CB and CNT addition have better piezoresistive sensitivity than the test samples with CB or CNT addition. Sample CB1CNT0.1 has the best piezoresistive sensitivity, with GFs of 9.71, 10.72 and 12.07 at 1%, 3% and 5% strain. It confirms that the two types of nano-carbon fillers of CB and CNT have a synergistic effect on the piezoresistive sensitivity of nano-carbon fillers/polymer composites from the experimental level. It is easy to see that a suitable combination of CB and CNT results in a sample with better piezoresistive sensitivity, which is consistent with the results of the numerical simulations. Certainly, due to the limitation of the number of samples, more detailed experimental results could not be obtained. This is exactly what needs to be complemented by numerical simulation methods.

In addition, it can also be found from Figure 10 and Figure 11 that the piezoresistive response of the composite test samples with single nano-carbon fillers doped shows a significant non-linear response relationship. The composite test samples with CB and CNT have some improvement in linearity. This suggests that the synergistic effect of CB and CNT also has a positive impact on the linearization of the piezoresistive response of the nano-carbon fillers/polymer composite.

**Figure 13:**
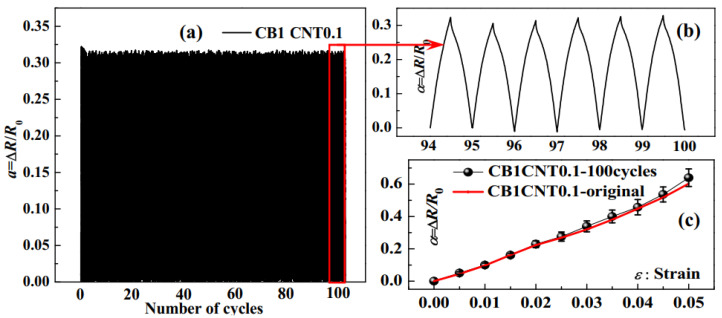
(**a**) The resistance change rate (Δ*R*/*R*_0_) under cyclic stretching–relaxing at 3.0% strain for 100 cycles; (**b**) The details of the 6 representative cycles extracted from the red pane of (**a**); (**c**) The tensile piezoresistive performance after 100 cycles test.

The sample CB1CNT0.1, with the best sensitivity among all tested samples, was selected for stability testing. The piezoresistive response results of the sample CB1CNT0.1 with the strain range of 0~3% and tensile rate of 5 mm/mi for 100 cycles are shown in Figure 13a. It can be seen that there is very little performance degradation at the beginning of cyclic loading, and the subsequent experimental results are relatively stable. Figure 13b shows the results of six cycles of the selected piezoresistive response. There is no significant attenuation, fluctuation or noise signal in the piezoresistive response curve. The test sample that has been subjected to 100 cycles of durability tests is again subjected to a single tensile experiment to test the piezoresistive response after the cycles, as shown in Figure 13c. The results indicate that the sample CB1CNT0.1 still has a stable piezoresistive response performance after the 100 cycles of the durability test.

## 4. Conclusions

In this work, we have systematically investigated the piezoresistive performance of CNT/CB/polymer composites via numerical simulation and experimental methods. The results show that the composite test samples doped with both CB and CNT have better piezoresistive sensitivity than the composite test samples doped with only CB or CNT. The synergistic enhancement effect of carbon nano-materials can be maximized by adjusting their types and contents appropriately. Effective numerical simulation algorithms can help researchers predict the type and amount of nano-carbon composites to be added at the optimal performance range.

## Data Availability

All data is available. Please contact the corresponding author for requests.
